# Calculating the individualized fraction regime in stereotactic body radiotherapy for non-small cell lung cancer based on uncomplicated tumor control probability function

**DOI:** 10.1186/s13014-019-1318-9

**Published:** 2019-06-20

**Authors:** Jia-Yang Lu, Pei-Xian Lin, Bao-Tian Huang

**Affiliations:** 1 Department of Radiation Oncology, Cancer Hospital of Shantou University Medical College, No.7 Raoping Road, Shantou, 515031 China; 2 Department of Nosocomial Infection Management, The Second Affiliated Hospital of Shantou University Medical College, 69 North Dongxia Road, Shantou, 515041 China

**Keywords:** Stereotactic body radiotherapy, Non-small cell lung cancer, Individualized fraction regime, Uncomplicated tumor control probability

## Abstract

**Background:**

To calculate the individualized fraction regime (IFR) in stereotactic body radiotherapy (SBRT) for non-small cell lung cancer (NSCLC) patients using the uncomplicated tumor control probability (UTCP, P^+^) function.

**Methods:**

Thirty-three patients with peripheral lung cancer or lung metastases who had undergone SBRT were analyzed. Treatment planning was performed using the dose regime of 48 Gy in 4 fractions. Dose volume histogram (DVH) data for the gross tumor volume (GTV), lung, chest wall (CW) and rib were exported and the dose bin was multiplied by a certain percentage of the dose in that bin which ranged from 1 to 200% in steps of 1%. For each dose fraction, P^+^ values were calculated by considering the tumor control probability (TCP), radiation-induced pneumonitis (RIP), chest wall pain (CWP) and radiation-induced rib fracture (RIRF). UTCP values as a function of physical dose were plotted and the maximum P^+^ values corresponded to the optimal therapeutic gain. The IFR in 3 fractions was also calculated with the same method by converting the dose using the linear quadratic (LQ) model.

**Results:**

Thirty-three patients attained an IFR using the introduced methods. All the patients achieved a TCP value higher than 92.0%. The IFR ranged from 3 × 10.8 Gy to 3 × 12.5 Gy for 3 fraction regimes and from 4 × 9.2 Gy to 4 × 10.7 Gy for 4 fraction regimes. Four patients with typical tumor characteristics demonstrated that the IFR was patient-specific and could maximize the therapeutic gain. Patients with a large tumor had a lower TCP and UTCP and a smaller fractional dose than patients with a small tumor. Patients with a tumor adjacent to the organ at risk (OAR) or at a high risk of RIP had a lower UTCP and a smaller fractional dose compared with patients with a tumor located distant from the OAR.

**Conclusions:**

The proposed method is capable of predicting the IFR for NSCLC patients undergoing SBRT. Further validation in clinical samples is required.

## Background

Stereotactic body radiotherapy (SBRT) has become a standard treatment alternative for patients with medically inoperable early stage non-small cell lung cancer (NSCLC), and for those refusing surgical resection [1–4]. Recent data have shown that SBRT provides outcomes that are equivalent to those of surgery [5–7].

Although SBRT for NSCLC has achieved encouraging outcomes, radiation-induced pneumonitis (RIP), chest wall pain (CWP) and radiation-induced rib fractures (RIRF) are common side effects for NSCLC patients undergoing SBRT. The occurrence of grade ≥ 2 RIP, grade ≥ 2 CWP and symptomatic RIRF ranged between 9.4 and 20.3% [8–15], 10.9 and 39% [16–20] and 12.2 and 17.0% [21–23], respectively. Therefore, to develop a method for calculating the individualized fraction regime (IFR) capable of maintaining tumor control probability (TCP) while lowering the risk of normal tissues by considering the tumor size and proximity to the organs at risk (OAR) is a problem to be solved. Recently, two independent studies utilized risk-adapted fraction regimes ranging from 3 to 8 fractions in SBRT treatment for lung cancer and achieved a low incidence of CWP [24, 25]. Unfortunately, the studies considered only the risk of CWP without considering of the tumor size or the toxicities of other OAR.

The current study aimed to develop a method to calculate the patient-specific fraction regime and to maximize the therapeutic gain for peripheral NSCLC patients by incorporating the uncomplicated tumor control probability (UTCP, P^+^) function.

## Methods

### Patient eligibility

Computed tomography (CT) simulation data for 33 patients previously diagnosed with primary stage I NSCLC or lung metastases were included in the study. The age of the patients ranged from 51 to 77 years. The basic characteristics of the patients are presented in Table 1.

**Table 1:** Basic characteristics of the patients

Patients	Gender	Age	Stage	GTV (cc)	PTV (cc)
1	M	73	ECa M	1.6	13.5
2	M	55	T1	1.6	27.6
3	F	55	T1	2	20.2
4	M	71	T1	2.4	19.1
5	M	71	T1	3.1	16.3
6	M	64	T1	3.3	23
7	M	62	T1	3.4	20
8	M	73	T1	3.5	18.3
9	M	68	T1	3.6	27.6
10	M	74	ECa M	3.7	50.3
11	F	59	T1	4	32.9
12	M	61	NPC M	4	26.8
13	F	72	T1	5.4	31.3
14	F	66	T1	6.2	83.8
15	F	71	T1	7	28.7
16	M	71	T1	7.7	36
17	M	64	T1	9.6	56
18	F	56	T1	9.7	63.5
19	M	51	SP M	10.1	48.7
20	M	68	T1	10.4	66.1
21	M	57	T1	10.9	40.9
22	M	75	T1	11.2	44.1
23	M	70	T1	11.6	40.8
24	M	74	T1	11.9	43.6
25	M	72	Lung M	14.9	64.3
26	M	71	T2	17.3	67.5
27	M	63	T2	20	73.7
28	M	72	T2	21	71
29	F	64	RC M	26.7	105.4
30	M	77	T2	26.7	95
31	M	77	ECa M	27.5	74.3
32	M	57	T2	44.4	119.7
33	M	51	T2	70.6	128.9

*Abbreviations: M* Male, *F* Female, *GTV* Gross target volume, *PTV* Planning target volume, *ECa M* Pulmonary metastasis from esophageal cancer, *NPC M* Pulmonary metastasis from nasopharyngeal carcinoma, *SP M* Pulmonary metastasis from soft palate cancer, *Lung M* Left lung metastases to the right lung, *RC M* Pulmonary metastasis from rectal cancer

### Immobilization and CT scanning

Patients were immobilized in the supine position with a vacuum bag (Medtec Medical, Inc., Buffalo Grove, IL) or a thermoplastic mask (Guangzhou Klarity Medical & Equipment Co., Ltd., Guangzhou, People’s Republic of China). All of the patients were simulated using a Brilliance Big Bore CT (Philips Brilliance CT Big Bore Oncology Configuration, Cleveland, OH, USA) under free breathing conditions. Ten-phase CT images were acquired at a 3-mm slice thickness during scanning using respiratory-correlated four-dimensional computed tomography (4DCT) via a Real-time Position Management System (Varian Medical System, Inc., Palo Alto, CA). Maximum intensity projection (MIP) and average intensity projection (AIP) images were reconstructed. The CT images, including the MIP and AIP images, were transferred to an Eclipse treatment planning system (Version 10.0, Varian Medical System, Inc., Palo Alto, CA) for target delineation, OAR contouring, treatment planning and plan evaluation.

### Target defining and OAR contouring

The internal target volume (ITV) was delineated by incorporating the gross tumor volume (GTV) on ten phases of the 4DCT scans under the pulmonary windows. To account for the set-up uncertainties and potential baseline tumor shift, a uniform 5 mm planning target volume (PTV) was created expanding around the ITV. For OAR contouring, the whole lung was limited to the air-inflated lung parenchyma, and the GTV and trachea/ipsilateral bronchus were excluded according to the Radiation Therapy Oncology Group (RTOG) 0915 report [26]. The CW was segmented from the corrected lung edges with a 2 cm expansion in the lateral, anterior, and posterior directions, excluding the lung volume and mediastinal soft tissue [16, 17, 27]. If the 2 cm expansion extended outside the body, then the contour extended only to the external patient surface. To avoid cumbersome delineation of the entire CW, we defined it within a 3 cm limit in the head-to-feet direction from the PTV [27]. To evaluate the incidence of RIRF after SBRT treatment, the rib that was within or closest to the target was delineated under a window level of 750 and a window width of 1400.

### Treatment planning

Dose regimes of 4 × 12 Gy were prescribed; 4 × 12 Gy represented 48 Gy in 4 fractions. The treatment was planned on the averaged 4DCT image using Eclipse treatment planning system (Version 10.0). All plans were designed on a TrueBeam LINAC with a 6 MV flattening filter free (FFF) photon beam and a maximum dose rate of 1400 MU/min. Treatment plans were created using dual partial arcs, preventing irradiation of the contralateral lung. The collimator angles for all plans were set to 30° and 330° to minimize the contribution of the tongue-and-groove effect to the dose. Optimization was performed using the progressive resolution optimizer (PRO_10028) algorithm. The objectives were adjusted to ensure a maximum dose higher than 120% of the prescribed dose center in the GTV. The dose was prescribed at 95% of the PTV covered by the prescription dose. Dose calculation was performed using the anisotropic analytical algorithm (AAA_10028) with a grid resolution of 1 mm while accounting for heterogeneity correction. All of the dose constraints and critical organ dose-volume limits should meet the criteria of the RTOG 0915 protocol and other publications [26, 28].

### Radiobiological models

The 3-year TCP data was predicted using the Liu et al. model [29] with the isocenter dose as a predictor. The model basically considers the tumor regrowth locally after radiation therapy, and thus can be applied to predict the TCP value as a function of follow-up time. The calculating formula and key modelling parameters were obtained from the original publication, with two respective sets of radiobiological parameters to predict the TCP data for stage T1 and T2 tumors. We employed the Wennberg et al. model [30] to predict the probability of 2-year grade ≥ 2 RIP. The model was Lyman-Kutcher-Burman (LKB) based and showed a dose–response relationship between RIP and the equivalent uniform dose (EUD) of the lung. The Din et al. model [18], a Cox proportional hazard (CPH)-based model, was applied to predict 2-year grade ≥ 2 CWP. The model used a combination of a normalized total dose of 99 Gy (NTD_99Gy_) and body mass index (BMI) for prediction. The beta coefficients of the model were acquired by taking natural logarithms of the hazard ratio (HR) values shown in Table 2 of the reference. The baseline hazard h_0_(t) value was obtained from the nomogram by privately contacting the author. The h_0_(t) value is approximately equal to 0.05 after careful measurement. The risk of 3-year RIRF was predicted using the Stam et al. model [23]. The model was a traditional normal tissue complication probability (NTCP) model in which the time to toxicity was taken into account. To calculate the probability of rib fracture within 3 years, we multiplied the NTCP value by a latency distribution, *f(τ)*, for the time to toxicity using the parameters described in Table 2 of the reference, for which a log-normal distribution is assumed. To estimate the probability of rib fracture within 3 years, the cumulative density function (CDF) was used to calculate the *f(τ)* value. The calculating process was performed using an in-house developed program on MATLAB 7.0 (MathWorks, USA).

**Table 2:** The IFR, TCP and UTCP values for the four patients with typical tumor characteristics

Parameters	Patient	3 fractions	4 fractions
IFR (Gy)	A	3 × 12.2	4 × 10.6
	B	3 × 11.6	4 × 10.0
	C	3 × 11.0	4 × 9.5
	D	3 × 10.8	4 × 9.2
TCP (%)	A	97.4	97.4
	B	94.7	94.5
	C	93.9	93.7
	D	94.9	94.6
UTCP	A	0.84	0.84
	B	0.79	0.79
	C	0.77	0.77
	D	0.73	0.74

*Abbreviations: IFR* Individualized fraction regime, *TCP* Tumor control probability, *UTCP* Uncomplicated tumor control probability

### Fraction regime individualization

The method described is based on maximizing the P^+^ function, where P^+^ = TCP·(1-NTCP) [31, 32]. In this study, P^+^ = TCP·(1-NTCP_lung_)·(1-NTCP_CW_)·(1-NTCP_rib_). The method investigates the P^+^ values when the physical dose changes. The dose volume histogram (DVH) data of the GTV (DVH_GTV_), lung (DVH_lung_), CW (DVH_CW_) and rib (DVH_rib_) were extracted from the treatment planning system at a resolution of 2 cGy. The dose bins of DVH_GTV_, DVH_lung_, DVH_CW_ and DVH_rib_ were then multiplied by a certain percentage of the dose in that bin which ranged from 1 to 200% in steps of 1%. Accordingly, 200 groups of DVH data were obtained. The TCP values of GTV (TCP_i_) and the NTCP values of the lung (NTCP_lung,i_), CW (NTCP_CW,i_) and rib (NTCP_rib,i_) from each dose fraction of the DVH were calculated separately.

**Fig. 1:**
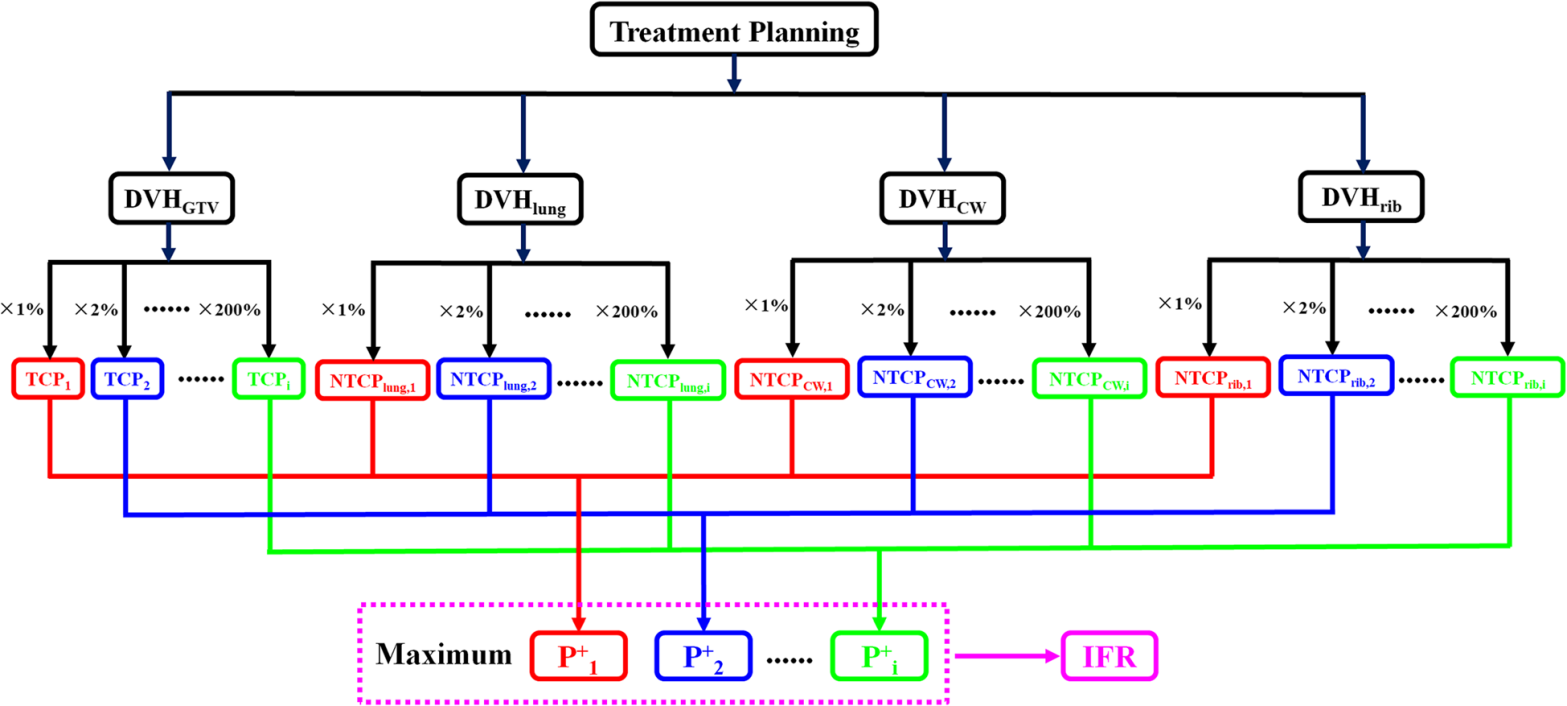
Process of fraction regime individualization. The P^+^ values considering the TCP and NTCP values of the lung, CW and rib for each dose bin of the DVH were calculated. The physical dose corresponding to the maximum P^+^ value is referred to as IFR. *Abbreviations:* DVH = Dose volume histogram; TCP = Tumor control probability; NTCP=Normal tissue complication probability; P^+^ = UTCP=Uncomplicated tumor control probability; IFR = Individualized fraction regime

The P^+^_i_ values for each of the 200 sets of DVH data were calculated. The maximum value for P^+^_i_ correspond to the optimal therapeutic gain. A flow chart of the fraction regime individualization is presented in Fig. 1. As fraction regimes of SBRT are often less than five fractions and single fraction regimes were reported to be unfit by the linear quadratic (LQ) model beyond a fractional dose of up to 20 Gy [33], the DVH_GTV_, DVH_lung_, DVH_CW_ and DVH_rib_ data were also converted to 3 fractions regimes using the LQ model. In other words, two groups of P^+^_i_ values, P^+^_i_ in 3 fractions (P^+^_i, 3f_) and P^+^_i_ in 4 fractions (P^+^_i, 4f_) were obtained.

## Results

### Clinical parameters

The tumor diameter ranges from 1.4–5.0 cm. The GTV and PTV ranges from 1.6–70.6 cc and 13.5–128.9 cc, respectively. Detailed clinical parameters for the patients are presented in Table 1.

### Individualized fraction regime

**Fig. 2:**
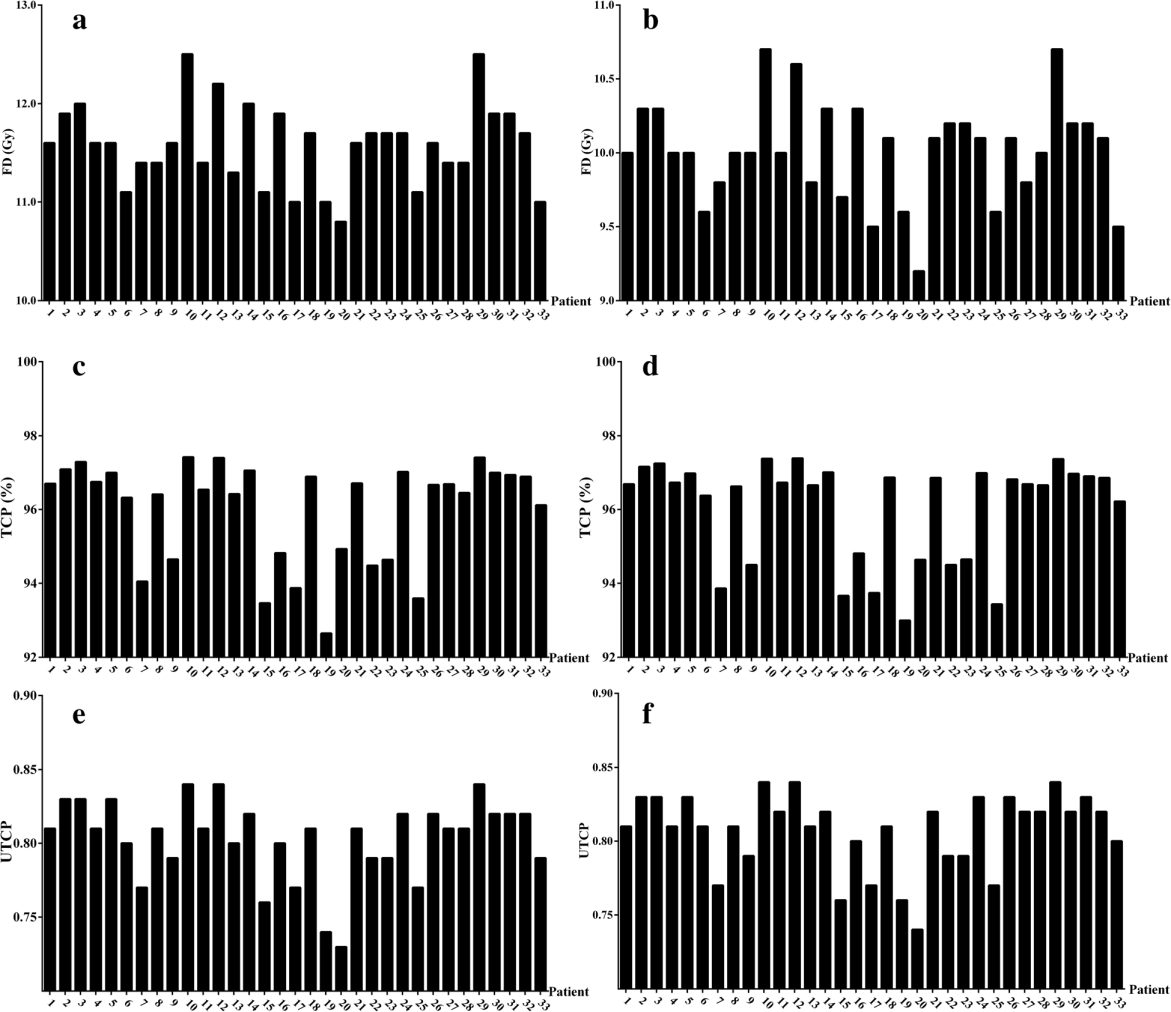
IFR, TCP and UTCP values across 33 patients. **a** IFR with 3 fractions; **b** IFR with 4 fractions; **c** TCP values with 3 fractions; **d** TCP values with 4 fractions; **e** UTCP values with 3 fractions; and **f** UTCP values with 3 fractions. *Abbreviations:* FD = Fractional dose of the IFR. TCP = Tumor control probability, UTCP = Uncomplicated tumor control probability

The IFR and corresponding TCP and UTCP values across the 33 patients are shown in Fig. 2. The figure shows that all the patients have patient-specific IFR, TCP and UTCP values. All the patients achieve a TCP value higher than 92.0%. The IFR ranges from 3 × 10.8 Gy to 3 × 12.5 Gy and from 4 × 9.2 Gy to 4 × 10.7 Gy for the 3 and 4 fraction regimes, respectively.

**Fig. 3:**
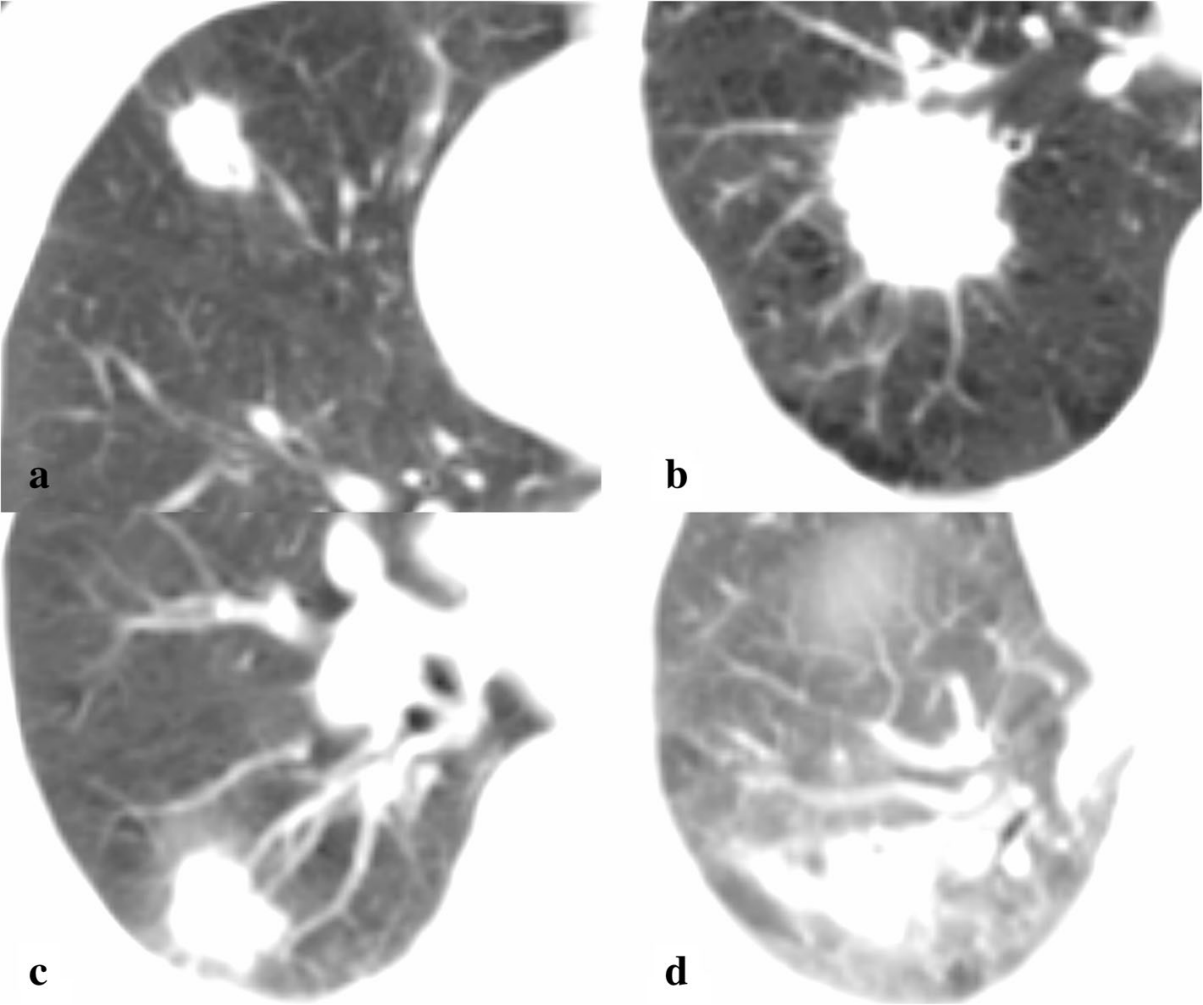
CT images of the four patients with typical tumor characteristics. **a** Patient with an off-CW small tumor; **b** Patient with an off-CW large tumor; **c** Patient’s tumor was adjacent to the CW; and **d** Patient at a high risk of RIP

**Fig. 4:**
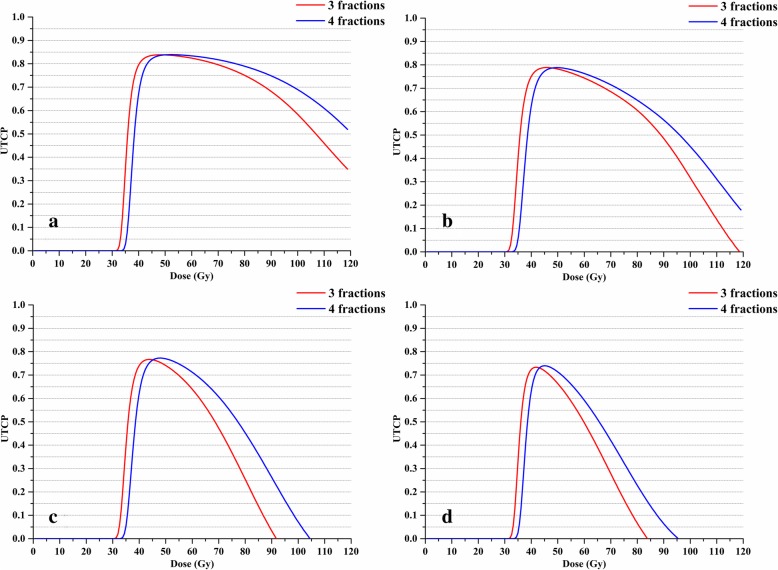
UTCP values as a function of physical dose for the four patients with typical tumor characteristics. The physical dose corresponding to the maximum UTCP value in the curve is referred to as the IFR. The IFR of 3 fractions and 4 fractions are displayed in red and blue, respectively. **a** Patient with an off-CW small tumor; **b** Patient with an off-CW large tumor; **c** Patient’s tumor was adjacent to the CW; and **d** Patient at a high risk of RIP. *Abbreviations:* UTCP = Uncomplicated tumor control probability

Four patients with typical tumor characteristics demonstrate that the method developed is personalized. Patient A has an off-CW small lesion (diameter of 1.9 cm and GTV of 3.4 cc) and patient B has off-CW large lesion (diameter of 3.7 cm and GTV of 26.7 cc). The tumor of patient C is adjacent to the CW and patient D has the largest target to normal lung volume ratio (PTV of 83.8 cc and the normal lung volume of only 1671.7 cc, predicted to be at high risk of RIP). A CT image of the four patients is shown in Fig. 3. Figure 4 shows the UTCP values as a function of physical dose for the four patients. The physical dose corresponding to the maximum UTCP value is referred to as the IFR, regardless of whether 3 or 4 fraction regimes are utilized. Table 2 shows that four patients with typical tumor characteristics possess IFR and individualized UTCP values while maintaining a TCP value higher than 93.0%. The UTCP values and fractional dose of the four patients in descending order are A > B > C > D. Patients with a large tumor has a lower TCP and UTCP and a smaller fractional dose than patients with a small tumor (patient B vs. patient A). Patients with tumor adjacent to the OAR (patient C) or at high risk of RIP (patient D) exhibits lower UTCP values and smaller fractional dose than patients with a tumor located distant from OAR (patient A and B).

## Discussion

In the current clinical practice of SBRT treatment for lung cancer, some “rigid” fraction regimes are commonly used. Clinical practice does not take into account tumor diversity, such as the size and proximity to normal tissues, which will lead to an underdose in some patients and to adverse side effects due to overdose in others. In this study, we first developed a method to calculate the IFR to avoid any underdose or overdose for NSCLC patients undergoing SBRT.

Efforts to search for the optimal dose of SBRT for stage I NSCLC are ongoing. Park et al. found that a biologically effective dose (BED) > 100 Gy was required to achieve a > 85% local control rate regardless of tumor size. The optimal dose for small tumors of < 3 cm appeared to be within a range below 150 Gy BED. The escalation of BED to high levels (> 150 Gy) might be required for patients with a tumor size larger than 3 cm [34]. Guckenberger et al. reported that doses of > 100 Gy BED to the CTV based on 4D dose calculation resulted in excellent local control rates [35]; Kestin et al. found a significant dose–response relationship for local control of NSCLC following image-guided SBRT with an optimal PTV_mean_ BED_10_ > 125 Gy [36]; Lee et al. observed that tumors ≤2 cm had no local recurrence regardless of dose, whereas for tumors > 2 cm, an escalated BED of approximately 150 Gy_10_ provided significantly higher local tumor control [37]; Koshy et al. concluded that higher doses (> 150 Gy BED) were associated with a significant survival benefit in patients with T2 tumors [38]; and Zhang et al. reported that a medium or medium to high BED (range, 83.2–146 Gy) for SBRT may currently be more beneficial and reasonable in stage I NSCLC [39]. However, the studies above drew inconsistent conclusions for the following reasons. First, the studies were derived from samples at various tumor stages and a wide variety of fraction regimes. Second, the studies mainly concentrated on local control data, and toxicity to the normal tissues was less considered. In other words, we believe that the optimal dose of SBRT for lung cancer is patient specific when the factors of tumor stage and proximity to the OAR are all considered. The so-called“one-size-fits-all” fraction regime does not exist.

The absolute values of LC and NTCP were partially dependent on the radiobiological models used. In the study, the regrowth model was employed to predict the TCP value for the following reasons: (1) It is the one and only TCP-predicting model to separate between stage T1 and T2 tumors. It was also proven to offer a better fit to the clinical data compared with the universal survival curve (USC) and the modified linear quadratic and linear (mLQL) models. (2) It is the only model to estimate local control for a certain follow-up time for primary lung cancer patients. (3) The model is highly in accordance with other published data in which the isocenter dose (also denoted as the maximum dose) was correlated with tumor local control [40, 41]. We used the Wennberg et al. model to calculate the risk of RIP because it uses bilateral lung exclusive of the GTV as the definition of lung volume, which is generally recognized in lung SBRT [8, 13, 15]. The Din et al. model and the Stam et al. model are unique models that calculate the incidence of CWP and RIRF. Moreover, 7 of the 33 patients in the study had pulmonary metastases from different primaries, and we assume a similar dose-response relationship between the primary and secondary lung tumors according to the results of Guckenberger et al. [42].

The applicability of the LQ model for local control modelling has been widely reported. Guckenberger et al. suggested that traditional LQ formalism could model patients with stage I NSCLC undergoing SBRT more accurately than LQ-L formalism based on 395 patients from 13 German and Austrian centers [40]. Shuryak et al. also found that the LQ model provided a significantly better fit to local control data for NSCLC than any of the models requiring extra terms at a high dose range [43]. Santiago analyzed 1975 patients and demonstrated that the LQ model was a robust method for predicting 3-year local control data [41]. Unfortunately, there is limited evidence on the validity of the LQ model for RIP prediction. Only Scheenstra et al. reported that the alpha/beta ratio of 1.3 Gy using the traditional LQ model was applicable for RIP prediction [44]. Whether the USC model characterized by an additional dose modification beyond a certain transitional dose (d_T_) is more suitable for modelling the RIP prediction has not been well established [45]. The lack of strong evidence for the applicability of the traditional LQ model for RIP prediction prompted us to use the Wennberg model, which is characterized by a USC model for converting the equivalent dose into 2 Gy fractions (EQD_2_) when the fractional dose of the DVH is greater than 5.8 Gy [30].

Although our study demonstrated that the method based on maximizing the P^+^ value is able to calculate the IFR in SBRT for lung cancer, there are some limitations. (1) We have to perform the treatment planning and export the dose data before calculating the IFR for each patient, which requires several hours to complete the process of fraction regime individualization. However, we may chart the IFR using the following variables when enough patients are analyzed because tumor size [29, 38], tumor-CW distance [46] and the PTV to total lung volume ratio [47] were widely reported to be correlated with LC, CWP, RIRF and RIP. (2) As the time points of TCP and NTCP data are inconsistent (2 year for CWP and RIP prediction and 3 year for TCP and RIRF prediction, we can’t determine whether the IFR was calculated in 2-year or 3-year follow up. (3) The study used the radiobiological models to calculating the P^+^ values; however, the correctness of the parameters is a bit questionable. Therefore, clinical validation is required to confirm the results.

## Conclusions

The proposed method based on maximizing the P^+^ value is able to predict the IFR in SBRT for lung cancer patients. However, clinical validation is required to confirm the results.

## Data Availability

The datasets used and/or analyzed during the current study are available from the corresponding author on reasonable request.

## Abbreviations

4DCT: Four-dimensional computed tomography
AAA: Anisotropic analytical algorithm
AIP: Average intensity projection
BED: Biologically effective dose
BMI: Body mass index
CDF: Cumulative density function
CPH: Cox proportional hazard
CT: Computed tomography
CWP: Chest wall pain
d_T_: Transitional dose
DVH: Dose volume histogram
EQD_2_: Equivalent dose in 2 Gy fractions
EUD: Equivalent uniform dose
FFF: Flattening filter free
GTV: Gross tumor volume
HR: Hazard ratio
IFR: Individualized fraction regime
ITV: Internal target volume
LKB: Lyman-Kutcher-Burman
LQ: Linear quadratic
MIP: Maximum intensity projection
mLQL: Modified linear quadratic and linear
NSCLC: Non-small cell lung cancer
NTCP: Normal tissue complication probability
OAR: Organs at risk
PRO: Progressive resolution optimizer
PTV: Planning target volume
RIP: Radiation-induced pneumonitis
RIRF: Radiation-induced rib fractures
RTOG: Radiation Therapy Oncology Group
SBRT: Stereotactic body radiotherapy
TCP: Tumor control probability
USC: Universal survival curve
UTCP: P^+^ uncomplicated tumor control probability

## References

[1] Baumann P, Nyman J, Hoyer M (2009). Outcome in a prospective phase II trial of medically inoperable stage I non-small-cell lung cancer patients treated with stereotactic body radiotherapy [J]. J Clin Oncol.

[2] Bonfili P, Di Staso M, Gravina GL (2010). Hypofractionated radical radiotherapy in elderly patients with medically inoperable stage I-II non-small-cell lung cancer [J]. Lung Cancer.

[3] Timmerman R, Paulus R, Galvin J (2010). Stereotactic body radiation therapy for inoperable early stage lung cancer [J]. JAMA.

[4] Xia T, Li H, Sun Q (2006). Promising clinical outcome of stereotactic body radiation therapy for patients with inoperable stage I/II non-small-cell lung cancer [J]. Int J Radiat Oncol Biol Phys.

[5] Solda F, Lodge M, Ashley S (2013). Stereotactic radiotherapy (SABR) for the treatment of primary non-small cell lung cancer; systematic review and comparison with a surgical cohort [J]. Radiother Oncol.

[6] Zhang B, Zhu F, Ma X (2014). Matched-pair comparisons of stereotactic body radiotherapy (SBRT) versus surgery for the treatment of early stage non-small cell lung cancer: a systematic review and meta-analysis [J]. Radiother Oncol.

[7] Zheng X, Schipper M, Kidwell K (2014). Survival outcome after stereotactic body radiation therapy and surgery for stage I non-small cell lung cancer: a meta-analysis [J]. Int J Radiat Oncol Biol Phys.

[8] Barriger RB, Forquer JA, Brabham JG (2012). A dose-volume analysis of radiation pneumonitis in non-small cell lung cancer patients treated with stereotactic body radiation therapy [J]. Int J Radiat Oncol Biol Phys.

[9] Baker R, Han G, Sarangkasiri S (2013). Clinical and dosimetric predictors of radiation pneumonitis in a large series of patients treated with stereotactic body radiation therapy to the lung [J]. Int J Radiat Oncol Biol Phys.

[10] Chang JY, Liu H, Balter P (2012). Clinical outcome and predictors of survival and pneumonitis after stereotactic ablative radiotherapy for stage I non-small cell lung cancer [J]. Radiat Oncol.

[11] Harder EM, Park HS, Chen ZJ, Decker RH (2016). Pulmonary dose-volume predictors of radiation pneumonitis following stereotactic body radiation therapy [J]. Practical Radiat Oncol.

[12] Matsuo Y, Shibuya K, Nakamura M (2012). Dose-volume metrics associated with radiation pneumonitis after stereotactic body radiation therapy for lung cancer [J]. Int J Radiat Oncol Biol Phys.

[13] Nakamura M, Nishimura H, Nakayama M (2016). Dosimetric factors predicting radiation pneumonitis after cyberknife stereotactic body radiotherapy for peripheral lung cancer [J]. Br J Radiol.

[14] Shi S, Zeng Z, Ye L, Huang Y, He J (2017). Risk factors associated with symptomatic radiation pneumonitis after stereotactic body radiation therapy for stage I non-small cell lung cancer [J]. Technol Cancer Res Treat.

[15] Yamamoto Takaya, Kadoya Noriyuki, Sato Yoshinao, Matsushita Haruo, Umezawa Rei, Kubozono Masaki, Ishikawa Yojiro, Kozumi Maiko, Takahashi Noriyoshi, Morishita Yohei, Katagiri Yu, Sato Kiyokazu, Ito Kengo, Takeda Ken, Jingu Keiichi (2018). Prognostic Value of Radiation Pneumonitis After Stereotactic Body Radiotherapy: Effect of Pulmonary Emphysema Quantitated Using CT Images. Clinical Lung Cancer.

[16] Dunlap NE, Cai J, Biedermann GB (2010). Chest wall volume receiving >30 Gy predicts risk of severe pain and/or rib fracture after lung stereotactic body radiotherapy [J]. Int J Radiat Oncol Biol Phys.

[17] Mutter RW, Liu F, Abreu A (2012). Dose-volume parameters predict for the development of chest wall pain after stereotactic body radiation for lung cancer [J]. Int J Radiat Oncol Biol Phys.

[18] Din SU, Williams EL, Jackson A (2015). Impact of fractionation and dose in a multivariate model for radiation-induced chest wall pain [J]. Int J Radiat Oncol Biol Phys.

[19] Murray L, Karakaya E, Hinsley S (2016). Lung stereotactic ablative radiotherapy (SABR): Dosimetric considerations for chest wall toxicity [J]. Br J Radiol.

[20] Woody NM, Videtic GM, Stephans KL (2012). Predicting chest wall pain from lung stereotactic body radiotherapy for different fractionation schemes [J]. Int J Radiat Oncol Biol Phys.

[21] Thibault I, Chiang A, Erler D (2016). Predictors of chest wall toxicity after lung stereotactic ablative radiotherapy [J]. Clin Oncol (R Coll Radiol).

[22] Asai K, Shioyama Y, Nakamura K (2012). Radiation-induced rib fractures after hypofractionated stereotactic body radiation therapy: risk factors and dose-volume relationship [J]. Int J Radiat Oncol Biol Phys.

[23] Stam B, van der Bijl E, Peulen H (2017). Dose-effect analysis of radiation induced rib fractures after thoracic SBRT [J]. Radiother Oncol.

[24] Coroller TP, Mak RH, Lewis JH (2014). Low incidence of chest wall pain with a risk-adapted lung stereotactic body radiation therapy approach using three or five fractions based on chest wall dosimetry [J]. PLoS One.

[25] Bongers EM, Haasbeek CJ, Lagerwaard FJ, Slotman BJ, Senan S (2011). Incidence and risk factors for chest wall toxicity after risk-adapted stereotactic radiotherapy for early-stage lung cancer [J]. J Thorac Oncol.

[26] Videtic GM, Hu C, Singh AK (2015). A randomized phase 2 study comparing 2 stereotactic body radiation therapy schedules for medically inoperable patients with stage I peripheral non-small cell lung cancer: NRG oncology RTOG 0915 (NCCTG N0927) [J]. Int J Radiat Oncol Biol Phys.

[27] Kong FM, Ritter T, Quint DJ (2011). Consideration of dose limits for organs at risk of thoracic radiotherapy: atlas for lung, proximal bronchial tree, esophagus, spinal cord, ribs, and brachial plexus [J]. Int J Radiat Oncol Biol Phys.

[28] Martin A, Gaya A (2010). Stereotactic body radiotherapy: a review [J]. Clin Oncol (R Coll Radiol).

[29] Liu F, Tai A, Lee P (2017). Tumor control probability modelling for stereotactic body radiation therapy of early-stage lung cancer using multiple bio-physical models [J]. Radiother Oncol.

[30] Wennberg BM, Baumann P, Gagliardi G (2011). NTCP modelling of lung toxicity after sbrt comparing the universal survival curve and the linear quadratic model for fractionation correction [J]. Acta Oncol.

[31] Agren A, Brahme A, Turesson I (1990). Optimization of uncomplicated control for head and neck tumors [J]. Int J Radiat Oncol Biol Phys.

[32] Pizarro F, Hernandez A (2017). Optimization of radiotherapy fractionation schedules based on radiobiological functions [J]. Br J Radiol.

[33] Ruggieri R, Stavrev P, Naccarato S (2017). Optimal dose and fraction number in SBRT of lung tumours: a radiobiological analysis [J]. Phys Med.

[34] Park S, Urm S, Cho H (2014). Analysis of biologically equivalent dose of stereotactic body radiotherapy for primary and metastatic lung tumors [J]. Cancer Res Treat.

[35] Guckenberger M, Wulf J, Mueller G (2009). Dose-response relationship for image-guided stereotactic body radiotherapy of pulmonary tumors: relevance of 4D dose calculation [J]. Int J Radiat Oncol Biol Phys.

[36] Kestin L, Grills I, Guckenberger M (2014). Dose-response relationship with clinical outcome for lung stereotactic body radiotherapy (SBRT) delivered via online image guidance [J]. Radiother Oncol.

[37] Lee S, Song SY, Kim SS (2018). Feasible optimization of stereotactic ablative radiotherapy dose by tumor size for stage I non-small-cell lung cancer [J]. Clin Lung Cancer.

[38] Koshy M, Malik R, Weichselbaum RR, Sher DJ (2015). Increasing radiation therapy dose is associated with improved survival in patients undergoing stereotactic body radiation therapy for stage I non-small-cell lung cancer [J]. Int J Radiat Oncol Biol Phys.

[39] Zhang J, Yang F, Li B (2011). Which is the optimal biologically effective dose of stereotactic body radiotherapy for stage I non-small-cell lung cancer? A meta-analysis [J]. Int J Radiat Oncol Biol Phys.

[40] Guckenberger M, Klement RJ, Allgauer M (2013). Applicability of the linear-quadratic formalism for modelling local tumor control probability in high dose per fraction stereotactic body radiotherapy for early stage non-small cell lung cancer [J]. Radiother Oncol.

[41] Santiago A, Barczyk S, Jelen U, Engenhart-Cabillic R, Wittig A (2016). Challenges in radiobiological modelling: can we decide between LQ and LQ-L models based on reviewed clinical nsclc treatment outcome data? [J]. Radiat Oncol.

[42] Guckenberger M, Klement RJ, Allgauer M (2016). Local tumor control probability modelling of primary and secondary lung tumors in stereotactic body radiotherapy [J]. Radiother Oncol.

[43] Shuryak I, Carlson DJ, Brown JM, Brenner DJ (2015). High-dose and fractionation effects in stereotactic radiation therapy: analysis of tumor control data from 2965 patients [J]. Radiother Oncol.

[44] Scheenstra AE, Rossi MM, Belderbos JS (2014). Alpha/beta ratio for normal lung tissue as estimated from lung cancer patients treated with stereotactic body and conventionally fractionated radiation therapy [J]. Int J Radiat Oncol Biol Phys.

[45] Park C, Papiez L, Zhang S, Story M, Timmerman RD (2008). Universal survival curve and single fraction equivalent dose: useful tools in understanding potency of ablative radiotherapy [J]. Int J Radiat Oncol Biol Phys.

[46] Nambu A, Onishi H, Aoki S (2013). Rib fracture after stereotactic radiotherapy for primary lung cancer: prevalence, degree of clinical symptoms, and risk factors [J]. BMC Cancer.

[47] Ueyama T, Arimura T, Takumi K (2018). Risk factors for radiation pneumonitis after stereotactic radiation therapy for lung tumours: clinical usefulness of the planning target volume to total lung volume ratio [J]. Br J Radiol.

